# More than just a metabolic regulator - elucidation and validation of new targets of PdhR in *Escherichia coli*

**DOI:** 10.1186/1752-0509-5-197

**Published:** 2011-12-14

**Authors:** Anna-Katharina Göhler, Öznur Kökpinar, Wolfgang Schmidt-Heck, Robert Geffers, Reinhard Guthke, Ursula Rinas, Stefan Schuster, Knut Jahreis, Christoph Kaleta

**Affiliations:** 1 Department of Genetics, University of Osnabrück, Barbarastraβe 11, D-49076 Osnabrück, Germany; 2 Helmholtz Centre for Infection Research, Inhoffenstraβe 7, D-38124 Braunschweig, Germany; 3 Institute of Technical Chemistry - Life Science, Leibniz University of Hannover, Callinstraβe 5, D-30167 Hannover, Germany; 4 Systems Biology/Bioinformatics Group, Leibniz Institute for Natural Product Research and Infection Biology - Hans Knöll Institute, D-07745 Jena, Germany; 5 Research Group Genome Analytics, Helmholtz Centre for Infection Research, Inhoffenstraβe 7, D-38124 Braunschweig, Germany; 6 Department of Bioinformatics, School of Biology and Pharmaceutics, Ernst-Abbe-Platz 2, Friedrich Schiller University of Jena, D-07743 Jena, Germany; 7 Research Group Theoretical Systems Biology, School of Biology and Pharmaceutics, Leutragraben 1, Friedrich Schiller University of Jena, D-07743 Jena, Germany

## Abstract

**Background:**

The pyruvate dehydrogenase regulator protein (PdhR) of *Escherichia coli *acts as a transcriptional regulator in a pyruvate dependent manner to control central metabolic fluxes. However, the complete PdhR regulon has not yet been uncovered. To achieve an extended understanding of its gene regulatory network, we combined large-scale network inference and experimental verification of results obtained by a systems biology approach.

**Results:**

22 new genes contained in two operons controlled by PdhR (previously only 20 regulatory targets in eight operons were known) were identified by analysing a large-scale dataset of *E. coli *from the Many Microbes Microarray Database and novel expression data from a *pdhR *knockout strain, as well as a PdhR overproducing strain. We identified a regulation of the glycolate utilization operon *glcDEFGBA *using chromatin immunoprecipitation and gel shift assays. We show that this regulation could be part of a cross-induction between genes necessary for acetate and pyruvate utilisation controlled through PdhR. Moreover, a link of PdhR regulation to the replication machinery of the cell via control of the transcription of the *dcw*-cluster was verified in experiments. This augments our knowledge of the functions of the PdhR-regulon and demonstrates its central importance for further cellular processes in *E. coli*.

**Conclusions:**

We extended the PdhR regulon by 22 new genes contained in two operons and validated the regulation of the *glcDEFGBA *operon for glycolate utilisation and the *dcw*-cluster for cell division proteins experimentally. Our results provide, for the first time, a plausible regulatory link between the nutritional status of the cell and cell replication mediated by PdhR.

## Background

The pyruvate dehydrogenase complex of *Escherichia coli *is encoded by the operon *pdhR-aceE-aceF-lpdA*. The first gene encodes the pyruvate dehydrogenase complex regulator (PdhR), which functions as a transcriptional regulator in a self-regulatory manner for this operon [1]. The multi-enzyme complex of the pyruvate dehydrogenase complex consists of 24 subunits of the pyruvate dehydrogenase (*aceE*), 24 subunits of the dehydrolipoate acetyltransferase (*aceF*), and 12 subunits of the dehydrolipoamide dehydrogenase (*lpdA*). This complex catalyses the formation of acetyl-CoA from pyruvate, which subsequently enters the TCA cycle [2]. Thereby the complex regulates the metabolic flux at the pyruvate node, which connects glycolysis and the TCA cycle.

The pyruvate dehydrogenase complex regulator (PdhR) belongs to the Gnt family of transcription factors [3] and is regulated by a pyruvate-sensing mechanism [1]. While PdhR represses the transcription of its target genes, the pyruvate-bound state of the regulator is not able to bind DNA. PdhR controls not only the transcription of the multi-enzyme complex of the pyruvate dehydrogenase complex, but also targets the *ndh *and *cyoABCDE *operons (genes encoding proteins for electron transport), which leads to the hypothesis that PdhR functions as a master regulator of genes involved in energy production and the following terminal electron transport from NADH to oxygen [4]. Furthermore, a connection between central metabolism and iron transport has been described by the regulation of the *fecABCDE *operon (genes for ferric citrate transporter) by PdhR [5]. The *tomB-hha *operon (antitoxin (TomB)-toxin (Hha)-module) [4] and the genes *hemL *(glutamate-1-semialdehyde aminotransferase) [4], *yfiD *(pyruvate formate-lyase subunit) [6], and *lipA *(lipoate synthase) [7] are also directly controlled by PdhR.

Since PdhR plays an important role in the control of metabolic flux, we aimed to identify further targets of this regulator. For this purpose we set out to identify potential targets of regulation by PdhR in a large-scale microarray dataset of *E. coli *from the Many Microbes Microarray Database [8]. Moreover, we constructed a *pdhR*-knockout and a PdhR-overproducing strain and studied their transcriptome on a variety of growth media. Following this analysis, we identified four potential binding sites of PdhR. Using chromatin immunoprecipitation (ChIP) in combination with quantitative PCR and gel shift assays we discovered that the *glcDEFGBA *operon (genes for glycolate utilisation, malate synthase) as well as the *mraZW-ftsLI-murEF-mraY-murD-ftsW-murGC-ddlB-ftsQAZ-lpxC *transcription unit (genes for proteins involved in cell division) are controlled by PdhR. Our results thus further underline the central importance of PdhR for the control of metabolism and its involvement in cell division by providing a link to the nutritional status of the cell. In consequence, we further support the notion that PdhR is an important component of the transcriptional regulatory network of *E. coli *[9].

## Results

**Figure 1:**
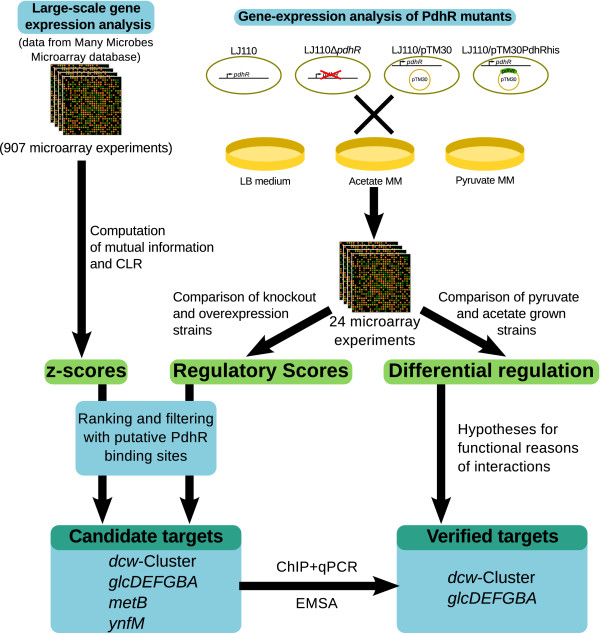
**Outline of the analysis**.

The outline of our study is presented in Figure 1.

### Construction of a *pdhR *deletion mutant and investigation of the phenotype on different media

The deletion mutant LJ110Δ*pdhR *was constructed as described in Methods. After confirming the genotype by PCR, the strains LJ110 and LJ110Δ*pdhR *as well as LJ110/pTM30 and LJ110/pTM30PdhRhis were investigated regarding their phenotypical growth behaviour. For this purpose strains were grown overnight and inoculated into various fresh media. Growth behaviour was observed by measuring the change in optical density. The growth rates μ [h^-1^] are given in Table 1.

**Table 1:** Effect of *pdhR *deletion and overexpression on growth

Strain	LB, μ [h^-1^]	MM glucose μ [h^-1^]	MM glycerol μ [h^-1^]	MM acetate μ [h^-1^]	MM pyruvate μ [h^-1^]
LJ110	1.29 +/- 0.01	0.59 +/- 0.07	0.42 +/- 0.01	0.17 +/- 0.02	0.21 +/- 0.07
LJ110Δ*pdhR*	1.25 +/- 0.06	0.20 +/- 0.019	0.19 +/- 0.02	0.07 +/- 0.02	0.22 +/- 0.00
LJ110/pTM30	1.28 +/- 0.02	0.60 +/- 0.00	0.41 +/- 0.01	0.14 +/- 0.03	0.20 +/- 0.02
LJ110/pTM30PdhRhis	1.26 +/- 0.01	0.53 +/- 0.00	0.43 +/- 0.01	0.13 +/- 0.03	0.19 +/- 0.01

Strains with different genotypes regarding *pdhR *were grown in rich medium (LB) and minimal medium (MM) supplied with glucose, glycerol, acetate or pyruvate as carbon source. The growth rate was determined in the mid logarithmic phase.

When grown in complex LB medium, neither the *pdhR *deletion mutant nor the PdhRhis overexpression strain differed in their growth behaviour from the parental strains. In contrast, when grown in minimal media supplemented with glucose, glycerol, or acetate as carbon source, a severe growth defect of the *pdhR *deletion mutant was observed. This phenotype emerged regardless of the carbohydrate source. Interestingly, this growth defect did not occur when cells were grown in minimal medium supplemented with pyruvate. Since the transcription factor PdhR is inactivated by pyruvate, the deletion is of no consequence under these conditions. Overproduction of the transcription regulator in minimal medium did not affect growth behaviour, as was observed in rich medium.

To get a more detailed insight into the effect of the chromosomal *pdhR *deletion and PdhR overproduction on the transcriptome, we analysed the gene-expression profiles of these strains under various growth conditions using microarrays.

### Elucidating further targets of PdhR in a systems biology approach

We used three different approaches to infer further targets of PdhR. Firstly, we identified putative targets of regulation by PdhR through the analysis of a large-scale gene-expression data set from the Many Microbes Microarray Database (M*^3D^*, [8]).

Secondly, we analysed microarray data from a PdhR overproducing and a *pdhR *knockout strain. Thirdly, we inferred putative phylogenetically conserved binding sites of PdhR on a genome scale using a previously described approach [7].

In the first approach, we determined an association score (z-score) indicating the significance of a regulation of each gene in the genome of *E. coli *by PdhR. These scores were determined from 907 gene-expression experiments, stored in M*^3D^*, using the context-likelihood of relatedness algorithm [5]. In the second approach, we obtained a regulatory score that corresponds to the strength of the effect of a knockout as well as an overproduction of PdhR on the expression of each gene during growth on three different media. In the third approach, we searched for putative phylogenetically conserved binding sites of PdhR in the upstream region of each gene of *E. coli*. We identified putative binding sites in the promoter regions of 363 operons containing 642 genes.

To identify further potential targets of PdhR, we sorted all genes in whose promoter region we identified a putative binding site of PdhR independently according to their z-scores and regulatory scores. The results of this analysis are displayed in Table 2. Thus, we found that particular genes belonging to the *dcw *cluster - which is important in cell division [10] - are the top-ranking targets according to their z-score. In contrast, genes of the *glcDEFGBA *operon, which are important in glyoxylate and glycolate utilisation, are enriched among the targets identified from the regulatory score. In addition to these two operons, we selected *ynfM *and *metB *as likely targets of a regulation by PdhR. We selected *ynfM *since it encodes a putative transport protein belonging to the major facilitory superfamily of transporters [11] and displays a marked increase of expression during growth on pyruvate, thereby being a potential pyruvate transporter (Additional File 1). Moreover we identified *metB*, which encodes an enzyme in methionine biosynthesis, as a putative target of PdhR.

**Table 2:** Identification of further targets of PdhR

Targets sorted by z-score				Targets sorted by regulatory score			
ID	Gene	Regulatory score	z-score	ID	Gene	Regulatory score	z-score
b1109	ndh*	2.913	7.803	b1109	ndh*	2.913	7.803
b0091	*murC***^$^**	-0.030	5.958	b0114	aceE*	2.250	5.866
b0115	aceF*	2.008	5.883	b4467	*glcF***^$^**	2.181	0.275
b0114	aceE*	2.250	5.866	b0115	aceF*	2.008	5.883
b0088	*murD***^$^**	-0.013	5.623	b2579	yfiD*	1.973	0.805
b0090	*murG***^$^**	-0.015	4.773	b2979	*glcD***^$^**	1.853	1.709
b0089	*ftsW***^$^**	0.073	4.664	b4467	*glcF***^$^**	1.163	0.275
b0084	*ftsI***^$^**	0.140	4.556	b2975	*glcA***^$^**	1.144	0.400
b0082	*mraW***^$^**	-0.056	4.297	b2977	*glcG***^$^**	1.056	2.315
b0087	*mraY***^$^**	0.086	4.229	b2601	*aroF*	1.051	1.723
b0125	*hpt*	0.002	4.149	b3828	*metR*	1.035	1.449
b3613	*envC*	-0.005	3.938	b2976	*glcB***^$^**	0.977	2.124
b0628	lipA*	-0.075	3.914	b1596	*ynfM*	0.927	0.007
b4052	*dnaB*	-0.018	3.864	b2600	*tyrA*	0.915	2.065
b0085	*murE***^$^**	0.167	3.659	b2505	*yfgH*	0.820	0.425
b0822	*ybiV*	0.284	3.653	b0333	*prpC*	0.729	0.021
b2683	*ygaH*	-0.077	3.628	b0331	*prpB*	0.713	0.024
b0436	*tig*	-0.260	3.620	b3939	*metB*	0.677	2.667
b4290	fecB*	0.315	3.561	b3547	*yhjX*	0.668	2.931
b0083	*ftsL***^$^**	0.119	3.536	b3426	*glpD*	0.659	0.904

Top-ranking genes with putative phylogenetically conserved binding sites of PdhR sorted according to z-scores (first four columns) and regulatory scores (last four columns). Previously known targets of PdhR are marked with "*" and new targets confirmed in this study with "$". Additionally to the new experimentally validated targets indicated in the above table, a regulation of *mraZ*, *murF*, *ddlB*, *ftsQ*, *ftsA*, *ftsZ *and *lpxC *by PdhR was confirmed as they are part of the operon that forms the *dcw*-cluster.

### Verifying four predicted binding sites by ChIP and qPCR

The analysis of our DNA microarray data combined with the data from databases, revealed new operons and pathways which might be regulated by PdhR. In the first experimental step four potential binding sites were chosen for further verification.

We tested the putative PdhR binding sites within the operator fragments of the genes *glcD *(encodes a subunit of the glycolate oxidase), *mraZ *(encodes a conserved protein in front of the *dcw *cluster), *metB *(encodes homocysteine transmethylase), and *ynfM *(encodes an unknown transporter protein) by chromatin immunoprecipitation (ChIP) and quantitative PCR (qPCR). Therefore, a culture which expresses his-tagged PdhR was grown in LB medium. The transcription regulator was cross-linked to chromosomal DNA. After cell lysis and shearing the DNA, DNA-repressor complexes were co-precipitated, the crosslinking reversed, and the DNA purified. This DNA was used as template DNA in the qPCR to compare the amounts of precipitated DNA fragments containing the described binding sites.

**Figure 2:**
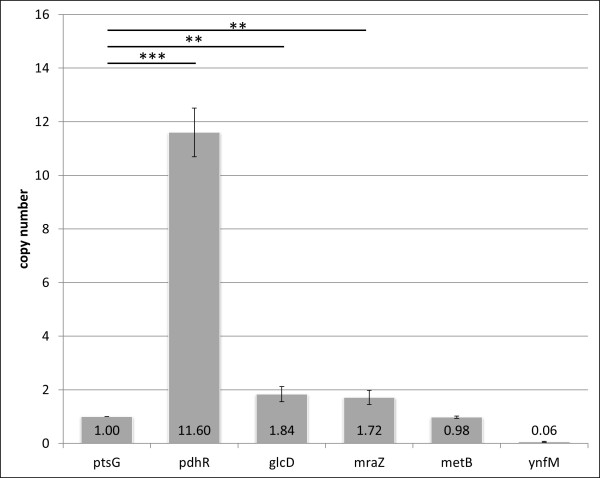
**Results of the ChiP and qPCR experiments**. His-tagged PdhR was crosslinked to DNA and purified. The co-precipitated DNA was analysed for the frequency of copies, which contain the putative binding sites of PdhR. The operator fragment of the not controlled *ptsG *gene was set as 1. The amount of DNA fragments which contain the known self-regulating pdhR binding site of *PdhR *was enriched to 11.6 times more copies compared to the negative control. The *glcD *and *mraZ *operator fragments occur to be 1.8 and 1.7 more abundant than the control fragment. The *metB *DNA fragment was detected with the same frequency as the control fragment and the *ynfM *binding site was observed to be less precipitated than the negative control. The data are mean values with standard deviations of three experiments. The statistical significance of the binding of PdhR to *pdhR*, *glcD *and *mraZ *if compared to the control by a One-Way-ANOVA test is indicated by asterisks (***: p-value < 0.001, **: p-value < 0.01).

Results are shown in Figure 2. For the tested primer pairs, the amount of PCR product for the *ptsG *gene that is not regulated by PdhR, served as a negative control PCR and was set as 1. DNA fragments of the known binding target for PdhR self-regulation in front of the *pdhR *gene were enriched by a factor of 11.6 in the assay. The qPCR revealed an enrichment of DNA fragments which contain the putative binding sites for PdhR in front of the genes *glcD *and *mraZ *by a factor of 1.84 and 1.72, respectively, that are statistically significant (Figure 2).

No enrichment was found for the putative PdhR target sequences in front of the genes *metB *(factor 0.98) and *ynfM *(factor 0.06). The applied growth conditions in complex rich medium might not be suitable for the detection of all PdhR-DNA interactions, although the overproduction of PdhRhis should facilitate binding, also to DNA fragments which might be bound with low affinity.

### Verifying three binding sites *in vitro *by gel shift assays

**Figure 3:**
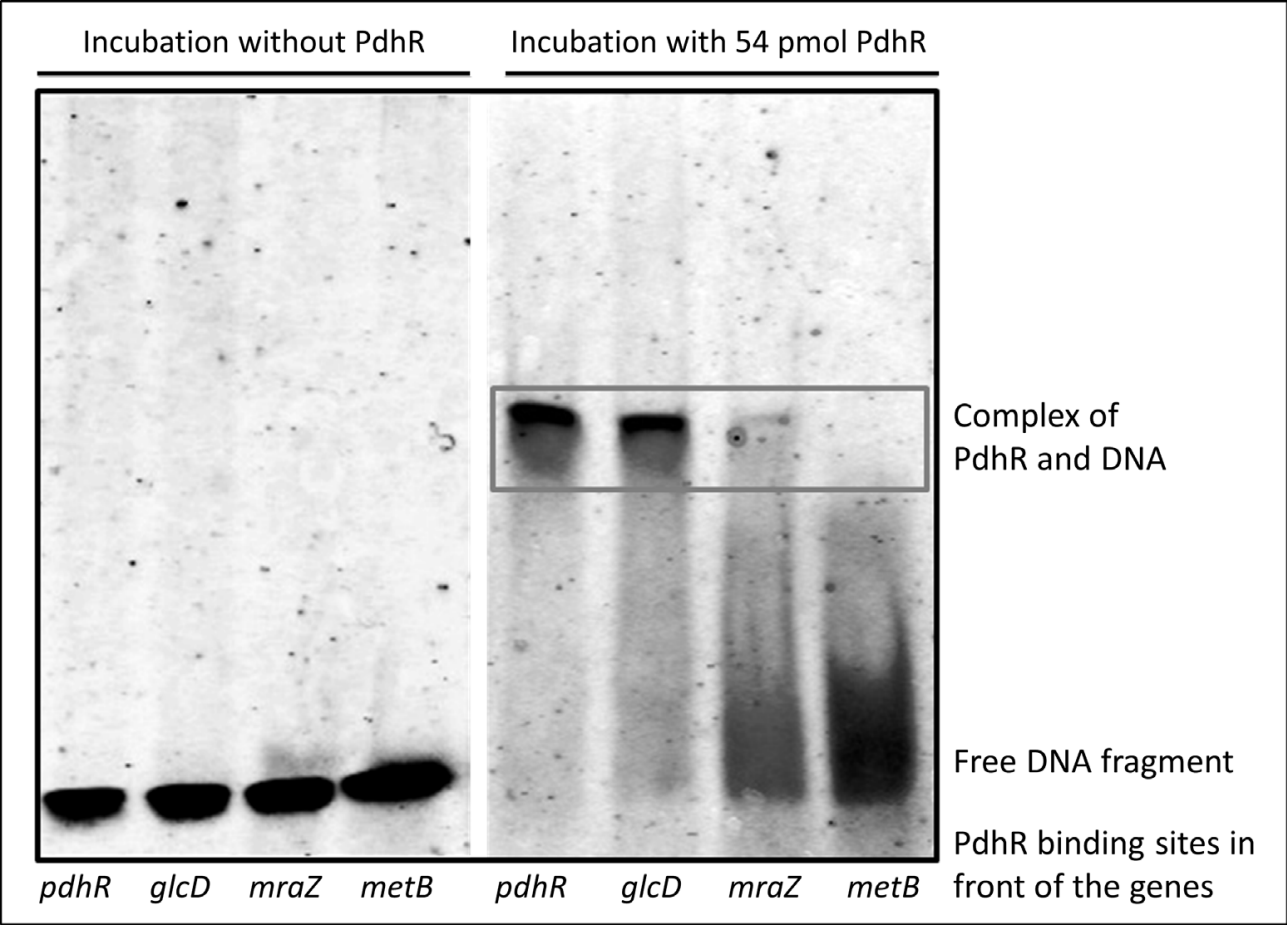
**Electrophoretic mobility shift assays**. The investigated binding sites in front of the genes *pdhR*, *glcD*, *mraZ*, and *metB *are displayed on the lower line. The left hand panel demonstrates an assay for 0.1 pmol of each DNA fragment without PdhR. For the assay shown on the right hand site, samples were incubated with 54 pmol purified PdhR prior to electrophoresis. Complex formation of regulator protein and DNA fragment leads to a shifted DNA-signal, which is assigned by the grey box. A strong PdhR-DNA complex is detected for the binding site in front of the *pdhR *gene for the known self-regulation. The same binding activity was monitored for the *glcD *binding site. Very weak affinity of PdhR was detected towards the *mraZ *binding site, nevertheless a small amount of shifted DNA was observed. No complex formation of PdhR with the binding site in front of *metB *gene was monitored.

In a second experimental step, three putative binding sites were further investigated. The binding activity of PdhR towards the binding sites in front of the genes *glcD*, *mraZ *and *metB *were also analysed by electrophoretic mobility shift assays. His-tagged PdhR was purified and incubated with fluorescence labelled DNA fragments. A complex formation of DNA and repressor protein leads to an electrophoretic retardation and thus a shifted fluorescence signal of the DNA fragment. The results are shown in Figure 3. The binding of purified PdhR to the known binding site of the operator region in front of the *pdhR *gene was observed. The same binding activity was detected for the region in front of the *glcD *gene, whereas the complex with the predicted binding site in front of the *mraZ *gene was much weaker. A complex with the DNA binding site of the *metB *gene was not detected in this *in vitro *approach.

### Assessing the global architecture of the PdhR regulon

Pertaining to the large number of processes regulated by PdhR we furthermore determined its mean expression level over the mean expression levels of all transcription factors of *E. coli *in the microarray data of M*^3D^*. Previously it was found that the number of targets of a transcriptional regulator correlates with its expression level [12]. The expression level of PdhR is close to the median of expression levels of all transcription factors. This suggests that while controlling distinct key cellular processes, PdhR does so by controlling a selected number of target genes rather than a large number of target genes like other global transcriptional regulators.

## Discussion

### Regulation of *glcDEFGBA *by PdhR

The *glcD *gene is the first gene of the transcription unit *glcDEFGBA*. This unit encodes the glycolate oxidase (GlcDEF), a small conserved protein of unknown function (GlcG), the malate synthase G (GlcB), and a glycolate transporter protein (GlcA). The operon is activated by GlcC during growth on glycolate and transcribed as a polycistronic message. The expression also depends on the integrative host factor (IHF) and is repressed by the respiratory regulator ArcA-P [13]. Glycolate and acetate are degraded via the common intermediate glyoxylate. Glyoxylate is an important intermediate of the central metabolism under conditions when acetate or fatty acids are the main carbon and energy source and is metabolised using the so-called glyoxylate bypass [14-16]. There are two isoenzymes, the malate synthases A and G (AceB and GlcB) that convert glyoxylate into malate. Both proteins belong to the acetate (AceB, encoded in the *aceABK *operon) or the glycolate/glyoxylate pathway (GlcB), respectively. Both operons are similarly controlled by the factors IHF and ArcA-P and can fulfil redundant roles via cross-induction [13] to avoid the toxic accumulation of glyoxylate.

**Figure 4:**
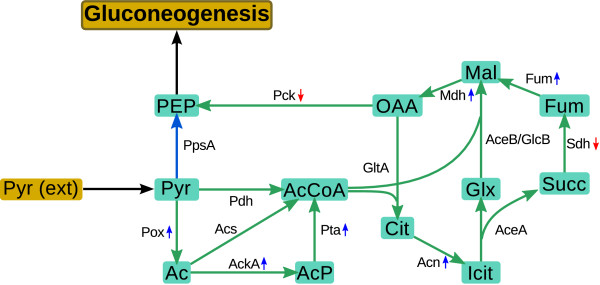
**Pathways involved in pyruvate metabolism**. Pyruvate (Pyr) can either be converted directly into phosphoenolpyruvate (blue arrows) or by utilising other pathways via the glyoxylate shunt and adjacent reactions (green arrows). Genes encoding enzymes that are on average more than twofold up- or downregulated in pyruvate grown cultures in comparison to acetate grown cultures are indicated by blue arrows (up-regulation in pyruvate grown cultures) and red arrows (down-regulation in pyruvate grown cultures). Enzyme abbreviations: AceA, isocitrate lyase; AceB/GlcB, malate synthase; Acn, aconitase; Acs, acetyl-CoA-synthetase; AckA, acetate kinase; Fum, fumarase; GltA, citrate synthase; Mdh, malate dehydrogenase; Pck, phosphoenolpyruvate carboxykinase; Pdh, pyruvate dehydrogenase; Pox, pyruvate oxidase; PpsA, phospoenolpyruvate synthase; Pta, phosphate acetyltransferase; Sdh, succinate dehydrogenase. Metabolite abbreviations: Ac, acetate; AcP, acetyl-phosphate; AcCoA, acetyl-CoA, Cit, citrate; Glx, glyoxylate; Icit, isocitrate; Succ, succinate; Fum, fumarate; Mal, malate; OAA, oxaloacetate; PEP, phosphoenolpyruvate; Pyr, pyruvate.

The commonly assumed route for pyruvate assimilation proceeds via the phosphoenolpyruvate synthase PpsA which allows gluconeogenesis from this compound. However, in a previous study it was found that the expression level of PpsA is suboptimal for growth on pyruvate [17]. This suggests that alternative routes may exist for pyruvate assimilation. The glyoxylate shunt could serve this purpose as it allows gluconeogenesis from the pyruvate derivative acetyl-CoA. This hypothesis is supported by the observation that the genes for PpsA and the glyoxylate shunt enzymes, AceA and AceB, did not show noticeable differences in expression during growth on acetate and pyruvate (Figure. 4). Moreover, we find that several genes which encode enzymes involved in the conversion of pyruvate into acetate are strongly upregulated in pyruvate grown cultures as compared to acetate grown cultures. Thus, a transcriptional regulation of the malate synthase GlcB, which is part of the *glcDEFGBA *operon and also part of the glyoxylate shunt, would allow the cell to control gluconeogenesis from pyruvate via the glyoxylate shunt (Figure. 4).

Extending the results of Pellicer and colleagues [13] who found a cross-induction of genes required for glycolate and acetate assimilation, our findings show that this cross-induction might also extend to genes activated during growth on pyruvate. This is supported by the regulation of the *glcDEFGBA *operon by PdhR and the finding, that many genes known to be upregulated during growth on acetate show no marked difference in expression during growth on acetate and pyruvate, while genes which encode enzymes that convert pyruvate into acetate are strongly upregulated during growth on pyruvate but not on acetate.

### Regulation of cell division by PdhR

**Figure 5:**
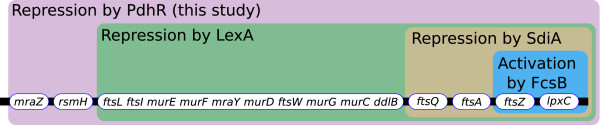
**Regulatory control of the *dcw*-cluster**. Transcription factors controlling the *dcw*-cluster. Sets of genes controlled by the same transcription factor are indicated by coloured boxes. Boxes with gene names indicate the transcriptional structure of the *dcw*-cluster. Every new box corresponds to a new transcription unit that is postulated to be transcribed until the distal end of the cluster. Adapted from EcoCyc [37].

MraZ is a protein of unknown function and is encoded in the transcription unit *mraZW-ftsLI-murEF-mraY-murD-ftsW-murGC-ddlB-ftsQAZ-lpxC *which represents the *dcw *cluster [10]. This unit encodes proteins involved in cell division and peptidoglycan biosynthesis. The expression of these genes is regulated in a highly sophisticated manner in order to time the cell division precisely (Figure 5). The gene products of this operon need to be synthesised at the correct time in the correct amount, and cell division has to be prevented when stress conditions prevail. It was shown that the *mraZ1p *promoter leads to transcription up to the *ftsW *gene [10]. The whole unit is also postulated to be transcribed in one message from the *mraZ1p *operator to the very distal gene *lpxC *[10]. Furthermore, there are many other promoter structures described or predicted in the cluster. Five transcription starts can be identified in the 5' region of the cluster. This regulation also includes repression by LexA binding to three identified SOS boxes. The transcriptional repressor LexA inhibits expression of genes involved in response to DNA damage and DNA replication inhibition in the so-called SOS response [18]. The repressor is inactivated by RecA-dependent cleavage after DNA damage [19].

At the 3' end of the cluster, six promoters have been identified that contribute to the correct timing and concentration of the *ftsZ *gene product which is essential for cell division [20]. Their expression is balanced by the promoters being constantly expressed gearbox promoters. These gearbox promoters are σ^s ^dependent promoters for growth rate regulation, inversely growth rate dependent-, and housekeeping promoters [21]. Additionally, an SdiA (**S**uppressor of the cell **d**ivision **i**nhibitor) dependent mechanism is integrated, which couples expression regulation to a quorum-sensing mechanism. Moreover, activation by the phosphorylated RcsB regulator (**R**egulator **c**apsule **s**ynthesis **B) **was shown.

For normal cell growth and correct division, a balance between the 5' and 3' encoded genes of the *dcw *cluster is required (for more details on *dcw *regulation see [10] and references therein).

Even though many regulatory mechanisms for the *dcw *cluster have already been identified, it is still not known how a link between the metabolic status of the cell and cell division is established. The pyruvate dehydrogenase complex regulator could fulfil this function, since it provides a cue to the nutritional status of the cell by sensing the pyruvate concentration. For instance, during growth on glucose, PdhR acts as a flux sensor for the glycolytic flux [22] that can serve as a proxy for the nutritional status of the cell. In the case of a high glycolytic flux, indicated by higher levels of pyruvate, the negative influence of PdhR on the *dcw *cluster is reduced whereas it is increased by a low glycolytic flux resulting in low pyruvate levels.

The influence of the metabolic status on cell division by PdhR is reflected by a high z-score in the large-scale analysis of microarray experiments. It is also experimentally supported by the fact that we detected a statistical significant enrichment of the *mraZ *operator binding site for PdhR by a factor of 1.7 in the *in vivo *assay. Up to this point we had only been able to detect very weak binding in our *in vitro *assays. This weak binding *in vitro *might be due to the complex regulation of the *dcw *cluster which most likely requires further interacting partners that we could not provide in our *in vitro *experiments.

## Conclusions

Taken together, we identified 22 new target genes contained in two operons controlled by PdhR using a bioinformatic and an experimental approach. The regulation of the *glcDEFGBA *operon and *glcB *in particular, as well as the comparison of gene-expression of acetate and pyruvate grown strains demonstrated that the metabolic state of the cell in both conditions is very similar. In particular, we found that in addition to the direct route to gluconeogenesis through the phospoenolpyruvate synthase, alternative pathways for the conversion of pyruvate to acetate appear to be activated and thus provide additional substrates for gluconeogenesis through action of the glyoxylate shunt.

**Figure 6:**
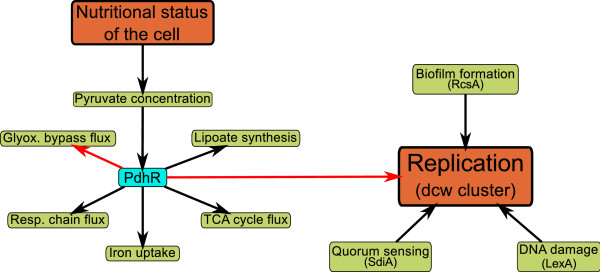
**A new view on the PdhR regulon**. PdhR acts as an important regulator of many cellular processes. The newly discovered regulation of the *dcw*-cluster by PdhR moreover provides an avenue through which the nutritional status of the cell can influence the replication process. Interactions drawn in red have been identified in this work.

Moreover, we have identified the *dcw*-cluster containing proteins required for cell division as a further regulatory target of PdhR. Through this regulatory interaction, we have established a plausible link between the nutritional status of the cell and cell replication which was not known to date. These results further support the hypothesis that PdhR is an important regulator of diverse processes of the cell by controlling a selected set of target genes rather than a large number of target genes like other global regulators (Figure 6). Thus, we have also demonstrated that, apart from being a central hub of metabolic fluxes, the pyruvate node exerts control on many aspects of bacterial physiology. In consequence, PdhR represents a promising target for further studies aimed at understanding central aspects of the interplay between metabolism and other cellular processes.

## Methods

### Experimental procedures

#### Bacterial strains, plasmids and media

The *Escherichia coli *K-12 strains LJ110 [23] and LJ110Δ*pdhR *(this study) were grown in Luria-Bertani broth (LB) or standard phosphate minimal medium [24] supplemented with 0.2% carbon source. When carrying the plasmids pTM30 [25] or pTM30PdhRhis [7], ampicillin was added in a concentration of 50 mg/liter. Cells were incubated at 37°C with shaking.

For overproduction of his-tagged PdhR the strain JM109 [26] was transformed with pTM30PdhRhis and gene expression induced with 1 mM IPTG. For construction of a *pdhR *deletion mutant, the strain BW25113 and the plasmids pKD4 and pKD46 [27] were used. If necessary, kanamycin was added in a concentration of 25 mg/liter.

For the deletion of the *pdhR *gene we followed the protocol of Datsenko and Wanner [27]. A 1728 bp PCR product was generated by standard PCR with the primer pair Pdhr_wanner+ (ATCCGCCAACCAAAACTCTCCGATGTGATTGAGCAG CAACTGGGTGTAGGCTGGAGCTGC) and Pdhr_wanner- (TTTCGTTGCTCCAGACGACGCAGAGAACGCTCA CGGCGGCTCTCTTCACGCATATGAATATCCTCCTTAG) and the plasmid pKD4 as template. The PCR product containing the kanamycin resistance cassette with flanking regions that are homologous to chromosomal sequences at the 5' and 3' end of the *pdhR *gene was purified with the Wizard DNA purification system (Promega), DpnI treated and further enriched by ethanol precipitation. In the next step it was transformed into BW25113 carrying pKD46. Transformants grown on LB plates with kanamycin were picked and the successful integration of the resistance cassette was verified by PCR using different primer combinations of k1 (CAGTCATAGCCGAATAGCCT), k2 (CGGTGC CCTGAATGAACTGC), kt (CGGCCACAGTCGATGAATCC), pdhr_downstream (TGATTTACAACATCTTCTGG) and pdhr_upstream (TGACTTCGGCAAGTGGCTTAAGAC). The chromosomal deletion of the *pdhR *gene in the BW25113 strain was then transduced into the strain LJ110 via P1 *vir *transduction generating the strain LJ110Δ*pdhR *following protocols by Arber [28] and Lengeler [29].

#### Determination of the growth rate

To determine the growth rate μ, cultures were grown overnight. The next day 10 ml medium was inoculated to an optical density_650/420 _= 0.1 and the OD was measured every hour. The growth rate μ was determined during the mid-logarithmic phase.

#### Sample preparation for microarray analysis

Cells were grown in LB- or minimal medium containing appropriate carbon sources and antibiotics. 1 × 10^9 ^cells (LB medium: OD_600 _= 1, 1 ml culture; minimal medium: OD_420 _= 0.5, 4 ml culture) were directly added to two volumes of RNAprotect Bacteria Reagent (Qiagen), mixed by shaking and incubated for 5 min at room temperature. Cells were pelleted by centrifugation (5,000 rpm, 10 min), the supernatant was removed and the pellet stored at -80°C.

#### DNA microarray hybridization and analysis

Total RNA was isolated from the cells using the protocol accompanying the RNeasy Mini Kit (Qiagen; Hilden, Germany). Quality and integrity of the total RNA was controlled on an Agilent Technologies 2100 Bioanalyzer (Agilent Technologies; Waldbronn, Germany). 200 ng of total RNA were applied for Cy3-labelling reaction using the MessageAmp II-Bacteria Kit according to supplier's recommendation (Ambion; Kaufungen, Germany). As a result of IVT (in vitro transcription) reaction using aminoallyl-dUTP antisense aRNA were generated and subsequently coupled with fluorescent dye Cy3. Cy3-labeled aRNA was hybridized to Agilent's 8 × 15 k *E. coli *microarray (Agilent Technologies; Waldbronn, Germany, AMADID 020097) for 16 h at 68°C and scanned using the Agilent DNA Microarray Scanner. Expression values (raw data) were calculated by the software package Feature Extraction 10.5.1.1 (Agilent Technologies; Waldbronn, Germany) using default values for GE1_105_Dec08 extraction protocol. Further data manipulation was applied according to section «Analysis of expression data from *pdhR *knockout and overexpressing strains». Gene-expression data has been submitted to Gene Expression Omnibus under the accession number GSE31333.

#### Chromatin immunoprecipitation

Chromatin immunoprecipitation based on a protocol from Jeremiah Faith (http://www.jeremiahfaith.com/open_notebook_science/ C.3 ChIP Protocols and [5]) was adapted with changes. 50 ml of LB medium in a 500 ml flask were inoculated with an overnight culture of LJ110/pTM30PdhRhis to an OD_650 _= 0.1 and grown for 30 min. The expression of the transcription factor was induced with 100 μM IPTG. At an OD_600 _= 1 15 ml culture were crosslinked using 37% formaldehyde in a final concentration at 1%. Crosslinking was performed by inverting the culture containing tube 10 times at room temperature. In the next step, cells were pelleted by centrifugation (3500 g, 10 min) and washed twice in cold PBS. The lysis of the cells and immunoprecipitation was performed using the μMACS™ His Isolation Kit (Miltenyi Biotec). The pellet was re-suspended in 1 ml of lysis buffer. 100 μl lysozyme (10 mg/ml) were added and incubated for 30 min on ice. Subsequently 10 μl 4-(2-aminoethyl)-benzensulfonylfluorid (AEBSF) (100 mM) and 10 μl RNAseA (10 mg/ml) were added and the sample was incubated another 30 min on ice. After cell lysis the DNA was sheared on ice by sonication using the Branson Digital Sonifier UNITS Models S-250D. The samples were sonified for 30 sec at 20% power and incubated on ice for one minute. Sonication was repeated four times. 900 μl of the sample were stored at -80°C. The remaining 100 μl were used for determining the sharing rate and incubated with 10 μl proteinase K (10 mg/ml) and 345 μl H_2_O_dd _at 65°C overnight to reverse crosslinking. The DNA was cleaned up using the GeneJet PCR purification Kit (Thermo Fisher) and run on a 1.5% agarose gel. The sharing range was determined to range from 200 bp to 1000 bp with an average size of DNA fragments around 500 bp. The immunoprecipitation was carried out using the μMACS Anti-His MicroBeads to isolate his-tagged PdhR protein from the sample following the manufactures' instructions. The 900 μl sample was thawed on ice and incubated with the magnetic beads, loaded onto the column and washed with buffers supplemented with the kit. The native DNA-protein-MicroBead complex was eluted in 100 μl TE buffer. The isolation of his-tagged PdhR protein was checked by analyzing 5 μl of the elution fraction by SDS-PAGE and Western blot analysis. Crosslinking was reversed by incubation of the remaining 95 μl of the elution fraction at 65°C overnight. 1 μl of proteinase K was added and the sample incubated at 45°C for two hours. In the next steps the DNA was separated from proteins and MicroBeads by a phenol/chloroform extraction. 0.5 ml phenol/chloroform was added and the sample mixed for 5 min. After one minute of incubation without mixing, the sample was mixed again for 2 min. The last two steps were repeated three times. Phase separation was carried out by a centrifugation step at 13000 rpm for 5 min. The DNA containing phase was transferred into a new cup and 1/10 volume of Na-Acetate (3 M, pH 6) and 1 ml ethanol were added. The sample was placed at -20°C overnight, centrifuged (13000 rpm, 30 min, 4°C), and washed in 80% ethanol. The pellet was air-dried and resuspended in 100 μl TE.

#### Quantitative PCR

Quantitative PCR was performed using the iCycler Thermal Cycler from BioRad. The qPCR was run in 25 μl reactions containing 12.5 μl Maxima™ SYBR Green qPCR Master Mix (Thermo Fisher), 2.5 μl primer+ and 2.5 μl primer- (10 pmol/μl), 5.5 μl H_2_O_dd _and 2 μl of the DNA which was co-precipitated, purified and concentrated. One DNA sample was used as template in six PCR reactions with each of the six primer pairs to compare the relative amount of PCR product. The primer pairs were chosen that they surround the putative binding site and give products of a length between 200 and 300 bps. The primers were checked in a standard endpoint PCR that they only amplify one product of the desired length. An annealing temperature of 51°C was determined to be suitable for all primer pairs. Therefore the reaction with all primer pairs could be run in the same qPCR. The primers are listed in Table 3. The results were analyzed with the qbase^PLUS ^software (Biogazelle).

**Table 3:** Primer pairs for qPCR

Binding site	Sequence 5'-3'	
PdhR_op_	CACAGTTTCATGATTTC	+
	GAGAGTGCCTTCGAG	-
GlcD_op_	CGGACCTCGTGCACAG	+
	GTGCCATCAGTACCG	-
MraZ_op_	AACGGTGATGACGATG	+
	GGTAGGCACTGATAAG	-
MetB_op_	GTGTAATGCACCTGTC	+
	AACCCGCTACGCACTG	-
YnfM_op_	ATGCAGCTCTTCCGC	+
	TTCTCAGTGTCGCTTG	-
PtsG_op_	GTCGGTAAATCGCTGATGCTGCC	+
	CAACAACTGCGGCCAGCGC	-

#### Purification of his-tagged PdhR

His-tagged PdhR was produced and purified as described earlier [7].

#### Gel shift assays

Assays were carried out as described previously [7]. All DNA probes were generated by annealing equimolar amount of fluorescence labeled primers (Thermo Fisher Scientific) of the known or predicted PdhR binding sites. The primers are listed in Table 4; binding regions are marked in grey.

**Table 4:** Fluorescence labeled primers for gel shift assays

Binding site	Sequence 5'-3'	Label
PdhR_op_	**GCCGAAGTCAATTGGTCTTACCAATTT**CATGTCTGTG	5'DY682
	CACAGACATG**AAATTGGAAGACCAATTGACTTCGGC**	5'DY782
GlcD_op_	CTATCTCTTT**AGCTACCGGTCAGACCATTTTTT**TTCCAGCTCT	5'DY682
	AGAGCTGGAA**AAAAAATGGTCTGACCGGTAGCT**AAAGAGATAG	5'DY782
MraZ_op_	TCGGTATGCC**TTGTGACTGGCTTGACAAGCTTTT**CCTCAGCTCC	5'DY682
	GGAGCTGAGG**AAAAGCTTGTCAAGCCAGTCACAA**GGCATACCGA	5'DY782
MetB_op_	AACGGCTATT**TGGGATTTGCTCAATCTATACGC**AAAGAAGTTT	5'DY682
	AAACTTCTTT**GCGTATAGATTGAGCAAATCCCA**AATAGCCGTT	5'DY782

### Bioinformatics procedures

#### Large-scale analysis of gene expression data from M*^3D^*

To elucidate further targets of PdhR we used data from 907 microarray experiments stored in M*^3D ^*[8]. We used the implementation of the context-likelihood of relatedness algorithm [5] provided in the R-package DTInfer [7] to determine potential targets of PdhR. In short, we computed the mutual information between the expression of *pdhR *and each gene of *E. coli *across the 907 microarray experiments. The significance of each of the mutual information values was estimated by computation of a z-score. This z-score is the square root of the sum of squares of two scores: the z^1 ^and the z^2^-score. For the mutual information value *I(i, j) *between the expression vectors of gene *i *and gene *j*, z^1 ^corresponds to the relative position of *I(i, j) *in the distribution of all mutual information values involving gene *i *and z^2 ^to the relative position of *I(i, j) *in the distribution of all mutual information values involving gene *j*. Since we were interested in regulatory targets of PdhR we subsequently discarded z-scores for all interactions not involving PdhR. By sorting genes according to their z-scores, we obtained a ranking of genes according to their likelihood to be regulated by PdhR. The z-scores of regulatory interactions involving PdhR are given in Additional File 1.

#### Analysis of expression data from *pdhR *knockout and overexpressing strains

To provide an independent line of evidence, we determined the gene expression of four different strains of *E. coli *on three different media. The four strains corresponded to *E. coli *LJ110, a *pdhR *knockout mutant (LJ110Δ*pdhR*), the parental strain carrying an empty plasmid (LJ110/pTM30) and the parental strain overproducing PdhR (LJ110/pTM30PdhRhis). These strains were cultivated on Luria-Bertani broth (LB), standard phosphate minimal medium supplemented with acetate and standard phosphate minimal medium supplemented with pyruvate. We obtained an overall 24 microarray experiments from two biological replicates of each of these cultivations as described above.

To analyse the quality of the microarray data, the raw gene-expression data were quantil-normalized using the package 'preprocessCore' of the Bioconductor Software [30]. The probes with low signal intensity were discarded for further analysis. In order to exclude samples that are not clearly attributable to their culture condition, the following quality check was performed. The high dimensional space of the gene expression data was mapped to a two- or three-dimensional space using the nonlinear Sammon projection method [31] implemented in the R package 'MASS' [32]. To detect possible outliers in the set of samples a model-based clustering approach using the R package mclust [33] was performed. This analysis identified three of the 24 microarrays that were not clearly attributable to culture conditions and were thus discarded from the subsequent analysis. To detect genes that were particularly affected by overexpression and knockout of *pdhR*, we determined for each gene *i *in each medium an average overexpression *o_i _*and an average knockout score *k_i _*as follows. Overexpression scores were determined by subtracting the log expression values of the strain carrying the empty plasmid from the overexpression strain. Knockout scores were obtained by subtracting the log expression value of the parental strain from the knockout strain. Due to the three discarded microarray experiments we thus obtained five knockout and four overexpression scores for each gene. For each gene the average knockout and average overexpression score was determined as average over the knockout and overexpression scores, respectively. Finally, we obtained a regulatory score over all microarrays by subtracting, for each gene, the overexpression score from the knockout score. Since PdhR is known to repress the transcription of most of its targets, we expect known targets of PdhR to have a high regulatory score in our experiments. The regulatory scores of all genes are given in Additional File 1.

#### Identification of candidates for experimental validation

To identify potential candidates for experimental validation of potential interactions we determined for each gene of *E. coli *whether we could identify a putative phylogenetically conserved transcription factor binding site of PdhR in its upstream region as described previously [7]. In brief, we aligned known binding sites of PdhR using the R-package *cosmo *[34] with the promoter region of each gene. If we thus identified a DNA sequence that resembled known binding sites of PdhR, we checked whether the corresponding region coincides with a part of the promoter known to be phylogenetically conserved upstream of genes in ten proteobacterial genomes [35,36]. We identified potential binding sites of PdhR in the promoter regions of 363 operons containing 642 genes. We ranked the genes in this list independently according to the z-score of a regulation by PdhR and according to the regulatory score obtained from our own microarray experiments. The top-ranking 20 candidates in either of both lists are displayed in Table 2.

#### Comparison of gene-expression between acetate and pyruvate grown cultures

Since no transporter for pyruvate is known in *E. coli *to date, we aimed to identify the corresponding gene(s) by comparison of gene-expression between acetate and pyruvate grown cultures. Thus, we computed average fold-changes between acetate and pyruvate grown cultures. The gene with the strongest overexpression possessing a putative phylogenetically conserved binding site of PdhR was *ynfM*. This gene encodes a transporter belonging to the major facilatory superfamily of transporters with yet unknown function. Moreover, *ynfM *showed a high regulatory score in the comparison of PdhR-knockout and overexpression strains (Additional File 1). These results led us to hypothesize that *ynfM *is a pyruvate transporter in *E. coli*. However, complementation studies in a mutant strain that does not grow on pyruvate minimal medium could not confirm these results (data not shown).

## Authors' contributions

AKG, ÖK and RoG conducted the experiments. CK, WSH, ÖK and RoG analyzed the microarray data. CK, KJ, SS, UR and ReG designed research. AKG, KJ, CK, ÖK and WSH wrote the paper. All authors read and approved the final manuscript.

## Supplementary Material

Additional file 1 **Results of gene-expression analysis**. Gene expression data, z-scores, regulatory scores and information on the position of detected binding sites. Click here for file
